# Positive relations between sexual quality of life and satisfaction with healthcare in women living with HIV and/or HCV: Results from a multicountry study

**DOI:** 10.1371/journal.pone.0278054

**Published:** 2023-01-20

**Authors:** Sara Rodriguez, Issifou Yaya, Ben Huntingdon, Ilona Juraskova, Marie Preau, Fatima Etemadi, Svetlane Dimi, Maria Patrizia Carrieri, Pascal Bessonneau, Olivier Chassany, Martin Duracinsky

**Affiliations:** 1 Unité de Recherche Clinique en Economie de la Santé (URC-ECO), Hôpital Hôtel-Dieu, AP-HP, Paris, France; 2 Patient-Centered Outcomes Research Unit, UMR 1123, INSERM, Université de Paris, Paris, France; 3 Clinical Psychology Unit, School of Psychology, The University of Sydney, Sydney, New South Wales, Australia; 4 Centre for Medical Psychology and Evidence-Based Decision-Making (CeMPED), The University of Sydney, Sydney, New South Wales, Australia; 5 Groupe de Recherche en Psychologie Sociale (GRePS), Université Lyon 2, Lyon, France; 6 Service de Médecine Interne, Hôpital Foch, Suresnes, France; 7 Aix Marseille Univ, Inserm, IRD, SESSTIM, Sciences Economiques & Sociales de la Santé & Traitement de l’Information Médicale, ISSPAM, Marseille, France; 8 Département de Médecine Interne et d’Immunologie Clinique, Hôpital Bicêtre, AP-HP, Le Kremlin-Bicêtre, France; Chinese University of Hong Kong, HONG KONG

## Abstract

**Introduction:**

The sexual quality of life is a neglected concern in women living with HIV (WHIV) or with HCV (WHCV), which can further be affected by their experience with stigma, social instability, fear of transmission and reduced access to treatment. The objective of this study was to identify sociodemographic, psychosocial, and behavioural factors associated with sexual quality of life (SQoL) in this study group.

**Methods:**

Between December 2017 and December 2018, PROQoL-Sex Life questionnaire was administered to 404 WHIV and WHCV in five countries. PROQoL-SQoL consists of four dimensions: positive sexual perception (*Psp*), stigma and social distress (*Sti*), soft sexual practices (*Sof*), sexual practices with a partner (*Sp)*, all of which were scored from 0 to 100 and considered as main outcomes, lower scores mean better sexual quality of life. Linear mixed effects models were used to evaluate the association with sociodemographic and psychosocial factors.

**Results:**

Of the participants analyzed, 191 were living with HCV, 180 with HIV and 33 with HIV and HCV, median age was 48. Among WHIV, a higher satisfaction with health care, and talking about sexuality with healthcare workers were associated with lower scores in all the dimensions of the SQoL, while psychoactive substance use was associated with lower scores of *Sti* and *Sof*. Moreover, higher satisfaction with health care, talking about sexuality with healthcare workers, and psychoactive substance use (except cocaine use) in WHCV were associated with lower scores in *Psp*, *Sti*, and *Sof*. Besides, cocaine use was associated with higher scores of *Sof*.

**Conclusion:**

This study highlighted strong relationship between the quality of health care, and psychoactive substance use (except cocaine) and the sexual quality of life in WHIV and WHCV in these five countries. These findings draw attention to the different interventions that can be proposed for improving the sexual quality of life.

## Introduction

HIV and HCV infections are significant public health concerns worldwide [1]. Overall, HIV-related mortality has decreased thanks to broad access to antiretroviral treatments (ART), however it have been higher among women than men [2]. Thus, HIV infection is increasingly considered a chronic pathology, and the assessment of quality of life is becoming essential in the follow-up of people living with HIV (PLWHIV). Moreover, even if advances in HCV infection treatments allow a complete cure. Complications, both hepatic and extrahepatic (including fatigue and depression), can persist with a considerable impact on the quality of life [3–5]. In high-income countries, several studies have shown that the quality of life of PLWHIV or people living with HCV (PLWHCV) remains comparable to that of the general population [6, 7]. However, the SQoL, one of the aspects of people quality of life, is often not explicitly explored in PLWHIV or PLWHCV.

WHO defines sexual health as a state of physical, mental and social well-being in relation to sexuality [8] as it plays an important role in the integral development of a human being. Despite this knowledge, the topic of sexual quality of life has been less in WHIV and WHCV which can be further be affected by their experience with stigma, social instability, substance use and reduce access to treatment [9, 10].

Sexual Quality of Life (SQoL) not only has a relevant impact on people’s wellbeing, but it is also associated with adherence to medication, sexual dysfunction such as decreased libido, anorgasmia and dyspareunia, other struggles are related to stigma, difficulties in finding a partner, fear of rejection and other psychological impacts [11, 12].

Several studies found that WHIV experience increasing sexual dysfunction either due to individual reasons including psychological factors and the use of ART [10, 13–15]. Both HIV and HCV infections can be controlled by prevention strategies, effective screening programs and access to treatments [16]. Since there are studies that have investigated sexual dysfunction, physical and physiological issues [9, 17, 18]. It became necessary to develop a quantitative questionnaire that assess SQoL in the context of HIV and HCV infections. Their SQoL could be addressed not only to promote an optimal SQoL but also to improve general quality of life and woman’s relationships. Moreover, sexuality is very often taboo and sometimes difficult to talk about it with healthcare professionals (HCP) during follow-up visits to HIV clinics, but this remained more pronounced among WHIV. The relationship between QoL and quality of healthcare was less frequently described in quality of life studies among PLWHIV and people living with HCV [12].

This study aimed to investigate the relations between individual (sociodemographic, behavioral and physiological) and structural factors associated with the SQoL, among WHIV and WHCV in five different countries.

## Methods

### Study design and participants

The data was obtained from a cross-sectional study validating the PROQoL-Sex Life among WHIV and WHCV treated at treatment centers in Brazil, Canada, Australia, the United States and France. Data were collected during a follow-up visit from December 2017 to December 2018 and the questionnaire was completed online. Inclusion criteria were: 18–75 years of age and living with HIV and/or hepatitis C and followed in one of the treatments centers involved in this study. Exclusion criteria were: Insufficient proficiency in the official language (English, French or Portuguese), presenting significant cognitive impairment or physiological disorder, hospitalization or having an acute infectious disease at the time of the study.

### Variable definitions

The main outcome in this study, the SQoL, was estimated using the validated PROQoL-Sex Life questionnaire [19] consisting of 20 items classified in fourth dimensions including: positive sexual perception (*Psp*), stigma and social distress (*Sti*), soft sexual practices (*Sof*), and sexual practices with a partner (*Sp*). For answers, a Likert-type scale from 1 (never) to 5 (always) was used, and dimensions were linearly transformed according to a standardized algorithm scored from 0 (best) to 100 (impaired).

The independent variables were: sociodemographic characteristics (gender, profession, level of education, marital status, life-style, origin, and sexual orientation), health status (health problems, treatment, and coinfections), mental health (depression, use of antidepressant treatment, psychiatric disease and feeling sad or anhedonia during the last four weeks), psychoactive substance use, sexual behaviors.

Infections related data were included: i) for HCV infection (viral load, time since HCV infection diagnosis, transmission route, treatment follow up and treatment of HCV infection), and ii) for HIV infection (CDC classification, transmission route, CD4 count, ART duration, and time since HIV infection diagnosis.

Behaviors related data from the SQR-12 questionnaire included prevention issues as knowledge about the risk of transmission and condom use. There were questions that explored satisfaction with health care support, and communication with health professional about sexuality. Sexual dysfunction among was assessed (Vaginal dryness, sexual desire, and pain during penetration).

### Statistical methods

For data cleaning and statistical analysis, R software was used (R Core Team 2013) along with several additional packages. A descriptive analysis was performed in order to describe the main characteristics of the study participants, some numerical variables such as age and duration of ART were converted into categorical variables, frequency and proportions were reported. In order to manage missing values, a simple imputation was performed using the K-Nearest Neighbor [20] method with the value of five. Then, bootstrap method stepwise linear regression approach was done using the package *caret* [21]. Variable selection for final linear regression model was done by stepwise AIC approach in order to find the subset of variables resulting in the best performing model, in a combination of forward and backward selection. The selected variables were used in a linear mixed effects model analysis, and fitted through the R function lme from the nlme [22] to assess the relationship between each score and variables. Each independent variable was included into the full model as fixed effects and the country was defined as random effect. Variance inflation factors were used to analyze the existence of potential collinearity problems, variance explained by the entire model was calculated using pseudo-R-square (MuMIn) and inter-class correlation (ICC) for checking reliability.

### Ethical consideration

Potential participants were informed orally about the study, voluntariness was underscored, before written consent was obtained. The study is registered on 02 February 2018 with identifier NCT03468673 in ClinicalTrial.gov. The project was approved by ethics committee in each country:

- Chair of the National Data Protection Commission (CNIL) in France (n°1837794v 0),
- Human Research Ethics Committee (HREC) of the South Eastern Sydney Local Health District in Australia (n° 15/056—HREC/15/POWH/157),
- Comitê de Ética Pesquisas (CEP) in Brazil (n°015/2014 –Codigo de Aceitação para Apreciação Ética (CAAE): 35564914.7.0000.5375).
- Canada (Institutional Review Board (IRB): 16152–15:07:3319-05-2017).

## Results

### Characteristics of study’s participants

In this study, 404 women were included, among them 180 WHIV, 191 WHCV and 33 women coinfected with HIV and HCV, distributed over five countries Brazil (N = 145), Canada (N = 24), Australia (N = 50), France (N = 123) and USA (N = 62). The Table 1 displays the characteristics of the participants. The median age of 47.8 years. The majority were married (44.4%), caucasian (57.6%), employed (43.5%) and with at least high school level (85.0%). The great majority women were heterosexual (92.8%) and 47.4% lived with a sexual partner with or without children and 38% lived alone or with their children.

**Table 1:** Characteristics of the study’s participants (N = 404).

Participants characteristics	N = 404	WHIV (n = 213)	WHCV (n = 191)	P-value
**Sociodemographics**				
**Age (years)**	**N = 404**	**n = 213**	**n = 191**	**0.004** ^1^
*Mean*	*47*.*8*	*45*.*7*	*50*.*2*	
**Occupation**	**N = 400**	**n = 209**	**n = 191**	**0.002** ^2^
*Employed*	*174 (43*.*5)*	*107 (50*.*2)*	*67 (35*.*1)*	
*Student*	*8 (2*.*0)*	*8 (3*.*8)*	*0 (0*.*0)*	
*Housewife*	*59 (14*.*8)*	*16 (7*.*5)*	*43 (22*.*5)*	
*Retired*	*71 (17*.*8)*	*30 (14*.*1)*	*41 (21*.*5)*	
*Unemployed*	*74 (18*.*5)*	*38 (17*.*8)*	*36 (18*.*8)*	
*Disable*	*14 (3*.*5)*	*10 (4*.*7)*	*4 (2*.*1)*	
**Education**	**N = 400**	**n = 210**	**n = 190**	0.372^2^
*Primary*	*60 (15*.*0)*	*32 (15*.*3)*	*28 (14*.*7)*	
*High school*	*233 (58*.*3)*	*116 (55*.*2)*	*117 (61*.*6)*	
*University*	*107 (26*.*7)*	*62 (29*.*5)*	*45 (23*.*7)*	
**Marital status**	**N = 401**	**n = 210**	**n = 191**	**0.042** ^2^
*Single*	*115 (28*.*7)*	*72 (34*.*3)*	*43 (22*.*5)*	
*Married*	*178 (44*.*4)*	*81 (38*.*6)*	*97 (50*.*8)*	
*Divorced*	*76 (18*.*9)*	*40 (19*.*0)*	*36 (18*.*8)*	
*Widow*	*32 (8*.*0)*	*17 (8*.*1)*	*15 (7*.*9)*	
**Living mode**	**N = 392**	**n = 206**	**n = 186**	**0.001** ^2^
*Alone*	*100 (25*.*5)*	*67 (32*.*5)*	*33 (17*.*7)*	
*Partner*	*115 (29*.*3)*	*52 (25*.*2)*	*63 (33*.*9)*	
*Children*	*49 (12*.*5)*	*31 (15*.*1)*	*18 (9*.*7)*	
*Children and partner*	*71 (18*.*1)*	*28 (13*.*6)*	*43 (23*.*1)*	
*Other living mode*	*57 (14*.*6)*	*28 (13*.*6)*	*29 (15*.*6)*	
**Origin**	**N = 403**	**n = 212**	**n = 191**	**<0.001** ^2^
*Caucasian*	*232(57*.*6)*	*110 (51*.*9)*	*122 (63*.*9)*	
*African*	*86 (21*.*3)*	*59 (27*.*8)*	*27 (14*.*1)*	
*Hispanic*	*45 (11*.*2)*	*17 (8*.*0)*	*28 (14*.*7)*	
*Asian*	*33 (8*.*2)*	*19 (9*.*0)*	*14 (7*.*3)*	
*Aboriginal*	*7 (1*.*7)*	*7 (3*.*3)*	*0 (0*.*0)*	
**Sexual orientation**	**N = 401**	**n = 210**	**n = 191**	0.108^2^
*Heterosexual*	*372 (92*.*8)*	*196 (93*.*3)*	*176 (92*.*1)*	
*Homosexual*	*11 (2*.*7)*	*8 (3*.*8)*	*3 (1*.*6)*	
*Bisexual*	*18 (4*.*5)*	*6 (2*.*9)*	*12 (6*.*3)*	
**Clinical outcomes**				
**Comorbidity**	**N = 400**	**n = 209**	**n = 191**	
*Diabetes*, *yes*	*44 (11)*	*19 (9*.*1)*	*25 (13*.*1)*	0.202^2^
*Cardiovascular*, *yes*	*46 (11*.*5)*	*28 (13*.*4)*	*18 (9*.*4)*	0.213^2^
*Psychiatrics*, *yes*	*79 (19*.*6)*	*34 (16*.*3)*	*45 (23*.*6)*	0.067^2^
*Depression*, *yes*	*110 (27*.*5)*	*50 (23*.*9)*	*60 (31*.*4)*	0.094^2^
**Mood**	**N = 399**	**n = 208**	**n = 191**	
Hopeless, yes	*177 (44*.*4)*	*89 (42*.*8)*	*88 (46*.*1)*	0.482^2^
Anhedonia, yes	*189 (47*.*4)*	*91 (43*.*8)*	*98 (51*.*3)*	0.120^2^
**Transmission route**	**N = 393**	**n = 203**	**n = 190**	**<0.001** ^2^
*Homosexual relation*	*7 (1*.*8)*	*2 (1*.*0)*	*5 (2*.*6)*	
*Heterosexual relation*	*219 (55*.*7)*	*193 (95*.*1)*	*26 (13*.*7)*	
*Injection*	*87 (22*.*1)*	*8 (3*.*9)*	*79 (41*.*6)*	
*Other transmission route*	*80 (20*.*4)*	*0 (0*.*0)*	*80 (42*.*1)*	
**Understand risk of HIV transmission** ^3^	**N = 210**	**n = 210**	NA	NA
*Not aware*	*17 (8*.*1)*	*17 (8*.*1)*		
*Neutral*	*19 (9*.*1)*	*19 (9*.*1)*		
*Moderate aware*	*75 (35*.*7)*	*75 (35*.*7)*		
*High aware*	*99 (47*.*1)*	*99 (47*.*1)*		
**HIV treatment** ^3^	**N = 183**	**n = 183**	NA	NA
*Nucleoside reverse transcriptase inhibitors (NRTIs)*	*4 (2*.*2)*	*4 (2*.*2)*		
*Non-nucleoside reverse transcriptase inhibitors (NNRTIs)*	*36 (19*.*7)*	*36 (19*.*7)*		
*Protease inhibitors (PIs)*	*68 (37*.*2)*	*68 (37*.*2)*		
*Integrase inhibitors (INI)*	*70 (38*.*2)*	*70 (38*.*2)*		
*Other*	*5 (2*.*7)*	*5 (2*.*7)*		
**HCV treatment** ^4^	**N = 147**	NA	**n = 147**	NA
*Ribavirine*	*101 (68*.*7)*		*101 (68*.*7)*	
*NS5A inhibitors*	*5 (3*.*4)*		*5 (3*.*4)*	
*Combine*	*2 (1*.*4)*		*2 (1*.*4)*	
*NS5B polymerase inhibitor*	*35 (23*.*8)*		*35 (23*.*8)*	
*NS3 inhibitor*	*2 (1*.*4)*		*2 (1*.*4)*	
*Other*	*2 (1*.*4)*		*2 (1*.*4)*	
**Viral load**	**N = 300**	**n = 194**	**n = 106**	**<0.001** ^2^
Detectable	41	7 (3.6)	34 (32.1)	
Undetectable	259	187 (96.4)	72 (67.9)	
**Talk to health professional about sexuality**	**N = 396**	**n = 209**	**n = 187**	0.106^2^
*No*	*205 (51*.*8*	*98 (46*.*9)*	*107 (57*.*2)*	
*Sometimes*	*71 (17*.*9)*	*43 (20*.*6)*	*28 (15*.*0)*	
*Yes*	*120 (30*.*3)*	*68 (32*.*5)*	*52 (27*.*8)*	
**Satisfied about healthcare care**	**N = 394**	**n = 207**	**n = 187**	0.056^2^
*Not satisfied*	*167 (42*.*4)*	*76 (36*.*7)*	*91 (48*.*7)*	
*Satisfied*	*141 (35*.*8)*	*82 (39*.*6)*	*59 (31*.*6)*	
*Very satisfied*	*86 (21*.*8)*	*49 (23*.*7)*	*37 (19*.*8)*	
**Behavioral**				
**Substance use**	**N = 401**	**n = 210**	**n = 191**	
*Alcohol*	*67 (16*.*7)*	*37 (17*.*6)*	*30 (15*.*7)*	0.608^2^
*Smoking*	*129 (32*.*2)*	*60 (28*.*6)*	*69 (36*.*1)*	0.106^2^
*Cocaine*	*19 (4*.*7)*	*8 (3*.*8)*	*11 (5*.*8)*	0.359^2^
*Cialis/Viagra*	*9 (2*.*2)*	*7 (3*.*3)*	*2 (1*.*0)*	0.123^2^
*Other drugs*	*39 (9*.*7)*	*17 (8*.*1)*	*22 (11*.*5)*	0.248^2^
*Sex stimulants*	*11 (2*.*7)*	*8 (3*.*8)*	*3 (1*.*6)*	0.170^2^
**Condom use**	**N = 395**	**n = 208**	**n = 187**	**<0.001** ^2^
*No*	*160 (40*.*5)*	*67 (32*.*2)*	*93 (49*.*7)*	
*Sometimes*	*53 (13*.*4)*	*27 (13*.*0)*	*26 (13*.*9)*	
*Yes*	*182 (46*.*1)*	*114 (54*.*8)*	*68 (36*.*4)*	
**Sexual dysfunctions**				
**Vaginal dryness**	**N = 389**	**n = 206**	**n = 183**	**<0.001** ^2^
*Frequently*	*137 (35*.*2)*	*44 (21*.*4)*	*93 (50*.*8)*	
*Sometimes*	*90 (23*.*1)*	*45 (21*.*8)*	*45 (24*.*6)*	
*Not frequently*	*162(41*.*6)*	*117 (56*.*8)*	*45 (24*.*6)*	
**Pain at penetration**	**N = 397**	**n = 210**	**n = 187**	0.868^2^
*Frequently*	*158 (39*.*8)*	*82 (39*.*1)*	*76 (40*.*6)*	
*Sometimes*	*59 (14*.*9)*	*33 (15*.*7)*	*26 (13*.*9)*	
*No frequently*	*180 (45*.*3)*	*95 (45*.*2)*	*85 (45*.*5)*	
**Sexual desire**	**N = 397**	**n = 209**	**n = 188**	0.361^2^
*Frequently*	*128 (32*.*2)*	*66 (31*.*6)*	*62 (33*.*0)*	
*Sometimes*	*153(38*.*5)*	*87 (41*.*6)*	*66 (35*.*1)*	
*Not frequently*	*116 (29*.*2)*	*56 (26*.*8)*	*60 (31*.*9)*	
**Worried during sex** ^3^	**N = 207**	**n = 207**		NA
*No worried*	*96 (47*.*3)*	*96 (47*.*29)*		
*Neutral*	*33 (16*.*3)*	*33 (16*.*26)*		
*Worried*	*52 (25*.*6)*	*52 (25*.*62)*		
*Very worried*	*22 (10*.*8)*	*22 (10*.*84)*		

^1^ = t-test;

^2^ = chi square test;

^3^ = Only for WHIV;

^4^ = Only for WHCV;

NA = non applicable

Depression and psychiatrics were reported respectively in 27.5% and 19.6% of the participants. Regarding transmission route, 95.1% of WHIV reported have acquired HIV in a heterosexual relation, while 41.6% of WHCV reported injection route. Regarding infection-related characteristics, the antiretroviral most used among WHIV was integrase inhibitors (38.2%). The majority of WHIV (96.4%) had undetectable HIV viral load (<50 copies/mL). Data on HCV treatment were available in three countries (Brazil, France and Canada). Among WHCV, 67.9% reported undetectable HCV viral load (Table 1).

About one third (32.2%) of the participants reported tobacco consumption, while 16.7% and 14.4% of the participants reported alcohol and drug use including cocain respectively. Regarding the satisfaction about the quality of health care support, 57.6% of the participants declared to be satisfied, while 48.2% reported having communication with HCP about sexuality. Consistent condom use during intercourse was reported in 46.1% of the participants (Table 1).

Concerning the sexual dysfunctions, 58.3% of the participants reported vaginal dryness, 54.7% reported pain during penetration, and 70.7% reported sexual desire (Table 1).

Compared to WHCV, WHIV were younger (p = 0.004), reported less married status (p = 0.042), reported more undetectable viral load (p<0.001), reported more consistent condom use (p<0.001) and reported less frequently vaginal dryness (p<0.001) (Table 1).

### PROQoL-Sex Life

The PROQoL Sexlife’s dimensions and their mean scores are shown in Table 2. The mean score of the PROQoL Sexlife’s dimensions varied from 26.2 in the *Sp* dimension to 69.8 in the *Sof* dimension among WHIV. While, it varied from 23.1 in the *Sp* dimension to 67.3 in the *Sof* dimension among WHCV (Table 2).

**Table 2:** PROQOL-Sex life dimensions.

PROQOL-Sex life dimensions*	WHIV, Mean	WHCV, Mean
**Positive sexual perception** (*Psp*)	49.5	45.1
**Stigma and social distress (*Sti*)**	41.6	37.4
**Soft sexual practices (*Sof*)**	69.8	67.3
**Sexual practice with partner** (*Sp*)	26.2	23.1

* the lower the score is, the better the SQoL is.

### Factors associated with PROQoL-Sex Life dimensions

Multivariable mixed linear regression results are shown in Table 3 for WHIV and in Table 4 for WHCV.

**Table 3:** Factors associated with PROQOL-Sex Life dimensions among WHIV (multivariable linear mixed effects regression).

SQOL dimensions (outcomes)*	Fixed effects				Random effect
	Participants’ characteristics	β	SE	p-value	
**Positive Sexual Perception (Psp)**	*Hispanic origin*	*-18*.*50*	*6*.*61*	*0*.*006*	***SD = 5*.*7*** ***ICC = 0*.*04*** ***R***^***2***^ = ***0*.*34***
	*Very satisfied about health care*	*-14*.*13*	*5*.*22*	*0*.*007*	
	*Cigarret use*	*-8*.*68*	*4*.*02*	*0*.*032*	
	*Talk about sex with health professional*	*-9*.*89*	*4*.*72*	*0*.*038*	
	*Worried during sex*: *neutral*	*15*.*60*	*5*.*29*	*0*.*004*	
	*Very worried during sex*	*18*.*41*	*6*.*47*	*0*.*005*	
**Stigma and social distress (Sti)**	*Marital status*: *widow*	*-15*.*57*	*5*.*67*	*0*.*007*	***SD = 3*.*03*** ***ICC = 0*.*01*** ***R***^***2***^ = ***0*.*43***
	*Other drugs use*	*-13*.*15*	*5*.*93*	*0*.*028*	
	*Hispanic origin*	*-12*.*47*	*5*.*48*	*0*.*024*	
	*Very satisfied about health care*	*-9*.*01*	*3*.*92*	*0*.*023*	
	*Cigarret use*	*6*.*82*	*3*.*38*	*0*.*046*	
	*Anhedonia mood*	*8*.*81*	*4*.*33*	*0*.*044*	
	*Worried during sex*	*11*.*05*	*4*.*07*	*0*.*007*	
	*CV disease*:	*14*.*26*	*4*.*43*	*0*.*002*	
	*Worried during sex*: *neutral*	*15*.*56*	*4*.*23*	*0*.*000*	
	*Very worried during sex*	*20*.*31*	*5*.*19*	*0*.*000*	
**Soft sexual practices (Sof)**	*Marital status*: *divorced*	*-24*.*17*	*5*.*54*	*0*.*000*	***SD = 4*.*4*** ***ICC = 0*.*18*** ***R***^***2***^ = ***0*.*51***
	*Understand about risk of HIV transmission*: *neutral*	*-24*.*05*	*9*.*82*	*0*.*016*	
	*Homosexual*	*-21*.*45*	*7*.*94*	*0*.*008*	
	*Sexual stimulants use*	*-19*.*26*	*8*.*28*	*0*.*022*	
	*Education level*: *university*	*-16*.*32*	*6*.*66*	*0*.*016*	
	*Employ*: *housewife*	*-16*.*01*	*5*.*86*	*0*.*008*	
	*Talk about sex with health professional*	*-13*.*68*	*4*.*02*	*0*.*001*	
	*Hopeless mood*	*9*.*91*	*4*.*04*	*0*.*016*	
	*African origin*	*13*.*48*	*4*.*20*	*0*.*002*	
	*Hispanic origin*	*15*.*84*	*5*.*24*	*0*.*003*	
	*Living with children*	*22*.*32*	*6*.*34*	*0*.*001*	
**Sexual practices with partner (Sp)**	*High awareness about risk of HIV transmission*:	*-44*.*62*	*19*.*33*	*0*.*028*	***SD = 0*.*009*** ***ICC = 0*.*04*** ***R***^***2***^ = ***0*.*56***
	*Very Satisfied about health care*	*-23*.*21*	*10*.*01*	*0*.*027*	
	*HIV treatment*: *non-nucleoside transcriptase inhibitor*	*-18*.*96*	*8*.*23*	*0*.*028*	
	*Age group*: *46*.*7–70*.*1*	*38*.*53*	*16*.*94*	*0*.*030*	

* the lower the score is, the better the SQoL is.

**Table 4:** Factors associated with PROQOL-Sex Life dimensions among WHCV (multivariable linear mixed effects regression).

SQOL dimensions (outcomes)*	Fixed effects				Random effect
	Participants’ characteristics	β	SE	p-value	
**Positive Sexual Perception (Psp)**	*Heterosexual transmission route (HCV)*	*-29*.*20*	*12*.*21*	*0*.*018*	***SD = 5*.*05*** ***ICC = 0*.*041*** ***R***^***2***^ = ***0*.*34***
	*Other HCV transmission route*:	*-25*.*71*	*12*.*31*	*0*.*038*	
	*Living with partner*	*-24*.*39*	*4*.*89*	*0*.*000*	
	*Injection transmission route (HCV)*	*-23*.*50*	*11*.*80*	*0*.*048*	
	*Living with children and partner*	*-21*.*90*	*5*.*48*	*0*.*000*	
	*Other drugs use*	*-17*.*00*	*5*.*93*	*0*.*005*	
	*Very satisfied about health care*	*-11*.*07*	*4*.*51*	*0*.*015*	
	*Condom use*	*-7*.*28*	*3*.*64*	*0*.*047*	
	*Depression*	*11*.*48*	*3*.*44*	*0*.*001*	
	*Age group*: *40–61*	*14*.*06*	*4*.*16*	*0*.*001*	
**Stigma and social distress (Sti)**	*Injection transmission route (HCV)*	*-30*.*19*	*9*.*78*	*0*.*002*	***SD = 3*.*02*** ***ICC = 0*.*17*** ***R***^***2***^ = ***0*.*38***
	*Heterosexual transmission route (HCV)*	*-25*.*64*	*10*.*34*	*0*.*014*	
	*Very satisfied about health care*	*-14*.*10*	*3*.*72*	*0*.*000*	
	*Asian origin*	*-11*.*48*	*5*.*47*	*0*.*038*	
	*Other drugs use*	*-10*.*64*	*5*.*04*	*0*.*036*	
	*Satisfied about health care*	*-7*.*69*	*3*.*34*	*0*.*023*	
	*Depression*	*10*.*03*	*3*.*18*	*0*.*002*	
	*Condom use*	*15*.*09*	*3*.*11*	*0*.*000*	
	*Take anticholesterol*	*15*.*36*	*7*.*33*	*0*.*038*	
	*Bactrim use as prevention treatment*	*23*.*64*	*11*.*10*	*0*.*035*	
**Soft sexual practices (Sof)**	*Other living mode*	*-22*.*63*	*6*.*60*	*0*.*001*	***SD = 0*.*02*** ***ICC = 0*.*18*** ***R***^***2***^ = ***0*.*36***
	*Asian origin*	*-22*.*16*	*6*.*34*	*0*.*001*	
	*Education level*: *university*	*-14*.*61*	*6*.*69*	*0*.*032*	
	*Education level*: *high school*	*-12*.*44*	*5*.*95*	*0*.*039*	
	*Alcohol consumption*	*-9*.*30*	*4*.*42*	*0*.*038*	
	*Satisfied about health care*	*-8*.*42*	*4*.*32*	*0*.*054*	
	*Age group*: *40–61*	*10*.*60*	*4*.*01*	*0*.*010*	
	*Cocaine consumption*	*14*.*42*	*6*.*86*	*0*.*039*	
	*Other HCV transmission route*	*22*.*19*	*10*.*96*	*0*.*046*	
**Sexual practices with partner (Sp)**	*Hispanic origin*	*-21*.*90*	*9*.*45*	*0*.*029*	***SD = 0*.*05*** ***ICC = 0*.*07*** ***R***^***2***^ = ***0*.*40***
	*Depression*	*20*.*21*	*9*.*48*	*0*.*043*	
	*Unemployed*	*22*.*94*	*10*.*67*	*0*.*041*	
	*Living with children*	*38*.*94*	*13*.*90*	*0*.*010*	

* the lower the score is, the better the SQoL is.

**Among WHIV** (Table 3), final models showed that being Hispanic, being very satisfied with health care, tobacco use and talking about sex with HCP were associated with lower scores in *Psp*. While being worried or neutral during sex was associated with impairment in *Psp* dimension.

Regarding the *Sti* dimension, widowed, Hispanic or WHIV satisfied with health care and those who reported drug use were less likely to experience stigma and social distress. While factors as cardiovascular disease, tobacco consumption, anhedonia and being worried or neutral during sex were associated with more stigma and social distress.

Being a housewife, university level education, divorced, homosexual orientation, use of sexual stimulants, talking about sex with HCP and being aware about HIV transmission were associated with lower score of *Sof*. While depression, African origin, Hispanic origin and living with children were associated with impaired *Sof* dimension.

WHIV who reported a higher satisfaction with health care, those who were aware of the risk of HIV transmission, and those who reported non-nucleoside transcriptase inhibitor treatment reported also lower score of *Sp*. Being older than 46 years was associated with a impaired *Sp*.

**Fig 1:**
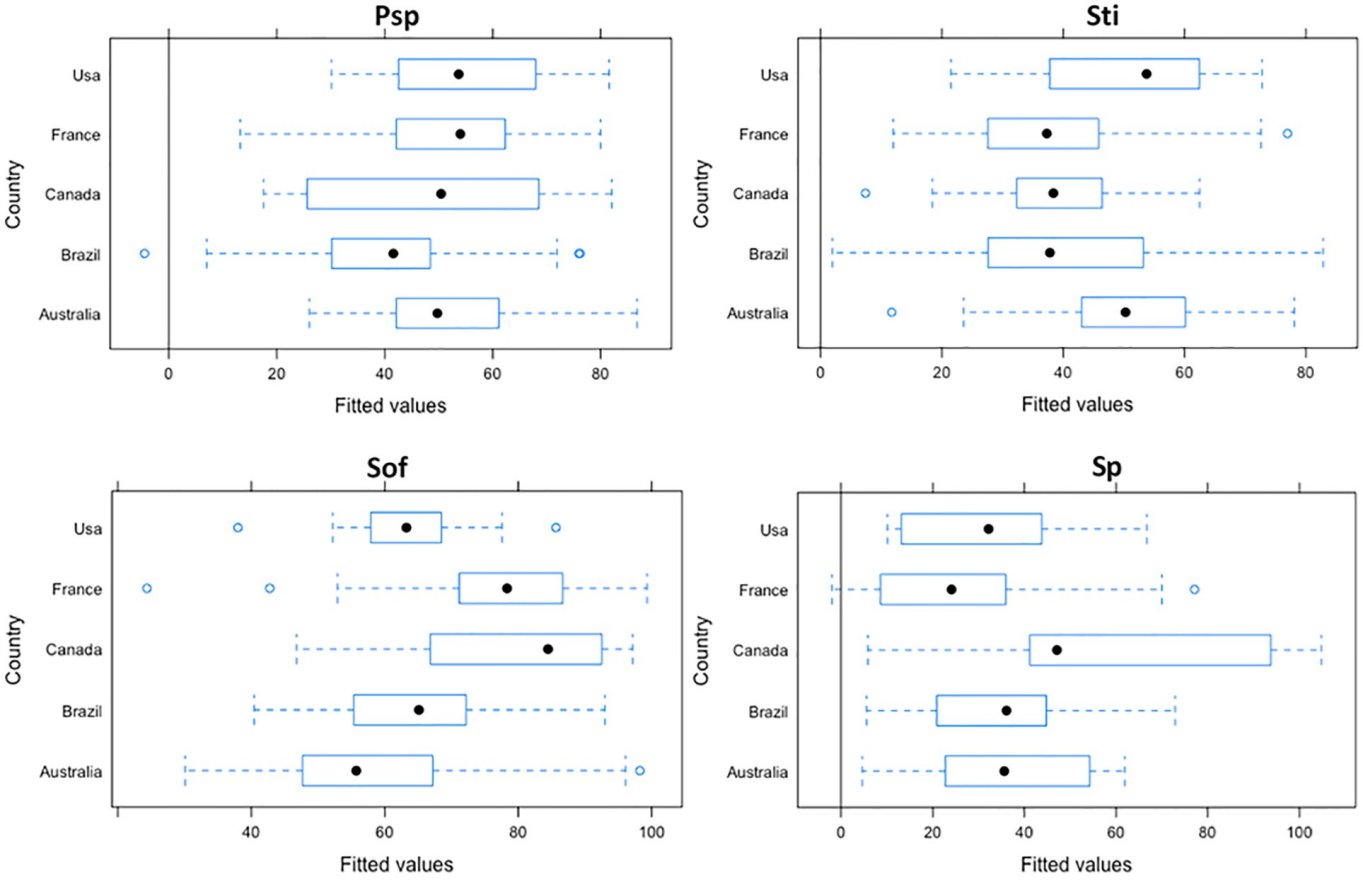
Variation of scores due to random effect among WHIV.

Adding country as random effect, Brazil in *Psp* dimension (-7), in *Sti* dimension (-2) and in *Sof* dimension (-5), while Australia (-7) in *Sp* dimension had a better outcome comparing with others countries. The Fig 1 showed how participants from five countries interact in PROQoL-Sex Life among WHIV.

**Among WHCV** (Table 4), being older than 40 years, and depression were associated with impaired *Psp* dimension. While living with a partner, drug use, heterosexual and injection HCV transmission route, higher satisfaction about health care, and condom use were associated with lower score of *Psp* dimension.

Regarding the second dimension *Sti*, Asian origin and drug use were associated with lower score of *Sti* dimension, in addition WHCV with a higher satisfaction about health care, and who reported injection route of HCV transmission were less likely to experience stigma. In contrast, depression, taking Bactrim as prevention treatment that reflects a lower CD4 count and consistent condom use were associated with impaired *Sti* dimension.

For soft sexual practices dimension, satisfaction about health care, asian origin, higher level of education, alcohol consumption, and living with other family members were associated with lower score of *Sof* dimension. Being older than 40 years was associated with impaired *Sof* dimension as well as cocaine consumption.

Factors associated with lower score of sexual practices with a partner was Hispanic origin, while depression, unemployment and living with children were associated with worse *Sp* dimension.

**Fig 2:**
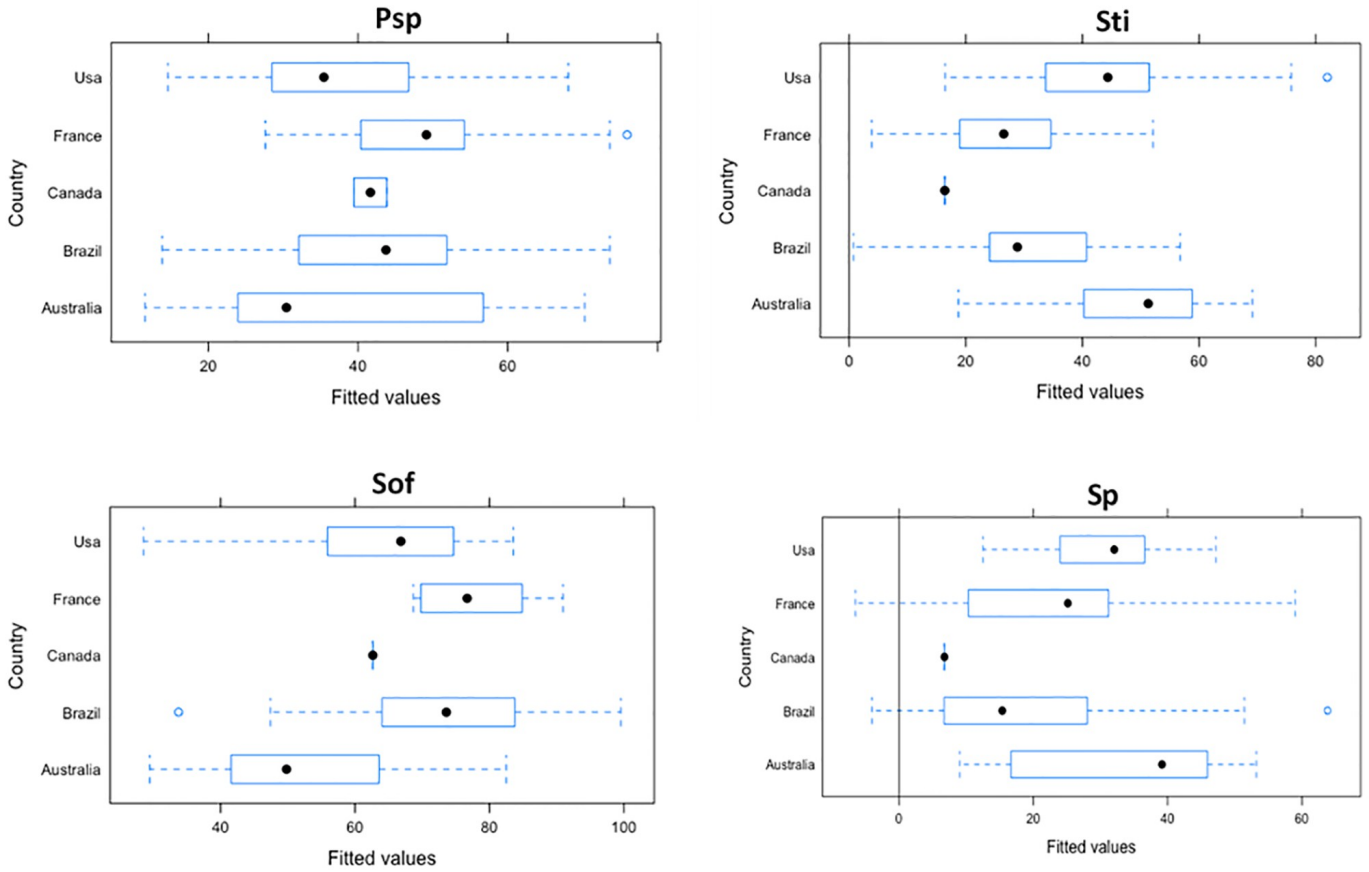
Variation of scores due to random effect among WHCV.

Adding Country as random effect, Brazil in *Psp* Dimension (-6), USA in *Sti* dimension (-0.93), in *Sof* dimension (-1) and Brazil in *Sp* dimension (-2.1); had a better outcome comparing with other others countries; the ICC was not bigger than 0.20, which means that variation explained by Country was not wide (Fig 2).

## Discussion

This multicountry study is one of the first studies that explore the SQoL among WHIV and WHCV. It showed a great variability through individual characteristics (sociodemographic and physiological) and structural factors (satisfaction with health system, including patient-HCP relationship and quality of health care). However, no significant variability between countries was observed.

Our analysis revealed globally higher scores of the SQoL, meaning a high sexual problems among WHIV and WHCV for most PROQoL-Sex Life’s dimensions. But, it was lesser for Sexual practices with partner dimension. In fact, the participants had commonly a better score for the dimension "Sexual practices with partner", which included preliminary and vaginal sexual relation aspects of sexual life. About four participants out of ten reported being married or living with a partner, with or without children, that may provide sexual satisfaction but also indirectly support in order to face stigmatization and other social rejection [23]. As highlighted in previous studies, sexual dissatisfaction was more reported in PLWHIV who did not have a regular sexual partner or were not living in couple [24, 25]. Studies either in WHIV or WHCV found that female sexual dysfunctions including desire, arousal, pain, orgasm, lubrication and satisfaction, were frequently reported [3, 10, 15, 26–28]. Many factors may affect female sexual function, including psychological and couple relationship factors [27]. Furthermore medical staff and social support, as mentioned in many other studies could be therefore needed to improve their global QoL, including the sexual dimensions [29–31]. For example, social support contributes to better global HRQOL by promoting healthy behaviors, avoiding stress and enhancing adherence to antiretroviral treatments [32–34].

We observed that, for WHIV the scores for all SQoL dimensions were slightly higher than those in WHCV. Even if this difference remains unclear, it should be noted that the characteristics of WHIV and WHCV included in this study were statistically different, in particular age, marital status, geographical origin but also consumption and sexual behavior. Besides, the duration of the HIV infection course and especially the exposure to antiretroviral may contribute to the impairment of some SQoL dimensions.

In addition, this study highlighted that interactions between patients and HCP could be beneficial to improve the SQoL in its global dimensions, both in WHIV and WHCV. Indeed, those who were satisfied with health care or those who talk about sex with HCP reported better SQoL scores. Although, there is few studies focused on the effect of interactions between WHIV or WHCV and HCW on the SQoL, perceived discrimination in healthcare settings has been found to be negatively associated with health-seeking behaviours, access HIV or HCV treatment, adherence and other clinical outcomes including viral load suppression, both in WHIV and WHCV [35–39]. Stigma against WHIV or WHCV in the healthcare settings is widespread in some countries and could be shown as for example HCP sitting far away from patients and or not touching them [35, 40, 41]. Besides, the role of HCP in the quality of global health care provided to WHIV or WHCV remains essential. This takes place through good communication allowing the establishment of an environment of mutual confidence, the good respect of the therapeutic indications and the instructions to the adherence ART. HCP may also take to ask their female HIV-positive patients, some essential questions related to sexual health, which could remove the stigma around discussing sex and normalize these discussions. In this environment, WHIV or WHCV may receive objective information about medical risks, preventive interventions for other diseases, psychological support and about safe sex practices.

Similar to previous studies in both PLWHIV and PLWHCV, psychoactive substance use, including alcohol, were associated with better score in the positive sexual perception, stigma and social distress and soft sexual practices dimensions [42–44]. This could be explained by the fact that the women who reported substance use usually are involved in high risk sexual behavior, which produces a strong sense of euphoria, physical agility and other feelings of mood elevation [45]. This positive association must be interpreted with great caution, especially since the substance use more often leads to an alteration of consciousness, and that this relative good perception may not really reflect participants experience. Besides, unsurprisingly in WHCV, those who reported cocain use had lower score of soft sexual practices.

This study also highlighted the influence of geographical origin on scores of several dimensions of SQoL. Hispanic participants reported lower scores of *Psp*, *Sti* and *Sp*, lower score of *Sof* were reported in Asian WHCV while higher score of *Sof* were reported in African and Hispanic WHIV. This could be mediated by cultural beliefs which could greatly vary across countries and regions. In addition, findings of this study have also shown that the differences between the five counties are not wide. It highlighted the importance of a support network among WHIV and WHCV, focused in all social determinants for each woman, as well the high importance that the quality of sexual activity plays as a determinant to wellbeing. Even though, sexual health is an essential element of overall health and well-being, the sexuality of women, including WHIV, is obscured by that of men in current patriarchal societies [46]. For example, in African society there exists several taboos about WHIV and they have no control about sexual practices [47]. More effort are needed in African communities to liberalize the voice of WHIV, including the expression and respect of their sexual right.

Non-nucleoside reverse transcriptase (NNRTI) was associated with lower scores in sexual practices with partner among WHIV. That could favor better adherence to ART overall well-being [48]. It is important to mention that this association remained unclear, and yet a previous study found a positive relation between NNRTI and a sexual satisfaction WHIV [49]. Also investigators suggest that this could be explained because NNRTI have a lower side effect burden than protease inhibitors.

This study had some limitations related to data collection, since the questionnaire was distributed in five countries; in three different languages there were variables that were not identical. Some countries had lower number of patients and especially variables as homosexual relationship status or taking drugs and Viagra, which was a limit for the study. In addition, clinical data was self-reported by the participants, and a recall bias may have affected the data, as well it was difficult to establish temporal relationships between variables. There are behavioral variables that could not be modified by interventions, such as the care or advice of healthcare workers. Our data are from a cross-sectional study and they cannot claim the determination of causality, so they are limited to describing the associations.

## Conclusion

This study revealed fairly higher scores in SQoL dimensions among WHIV and WHCV in these five countries. In addition the quality of health care, and psychoactive substance use (except cocaine) were associated with the quality of sexual life in WHIV and WHCV. This give us a wide vision about the necessity to assess WHIV and WHCV patients not only clinical analysis but also all determinants, in order to improve their quality of sexual life.
